# Socioeconomic disparities in *Plasmodium falciparum* infection risk in Southern Malawi: mediation analyses

**DOI:** 10.1038/s41598-024-78512-1

**Published:** 2024-11-08

**Authors:** Solomon T. Wafula, Oumou Maiga-Ascofare, Nicole S. Struck, Don P. Mathanga, Lauren M. Cohee, Jürgen May, Dewi I. Puradiredja, Eva Lorenz

**Affiliations:** 1 Department of Infectious Disease Epidemiology, Bernhard Nocht Institute for Tropical Medicine, Hamburg, Germany; 2 Department of Disease Control and Environmental Health, School of Public Health, Makerere University, Kampala, Uganda; 3 German Center for Infection Research (DZIF), Partner Site Hamburg-Borstel-Luebeck-Riems, Hamburg, Germany; 4 School of Global and Public Health, Kamuzu University of Health Sciences (KUHeS), Private Bag 360, Blantyre 3, Chichiri, Malawi; 5 Malaria Alert Centre (MAC), Kamuzu University of Health Sciences (KUHeS), Private Bag 360, Blantyre 3, Chichiri, Malawi; 6 Malaria Research Program, Center for Vaccine Development and Global Health, University of Maryland School of Medicine, Baltimore, USA; 7 Liverpool School of Tropical Medicine, Liverpool, UK; 8 University Medical Center Hamburg-Eppendorf (UKE), Hamburg, Germany

**Keywords:** Malaria, Plasmodium Falciparum, Socioeconomic position, Mediation, Counterfactual framework, Malawi, Diseases, Health care, Medical research, Risk factors

## Abstract

This study investigated the mediators of the association between socioeconomic position (SEP) and *Plasmodium falciparum (Pf)* infection in Southern region of Malawi. We utilized data from the 2014 International Center of Excellence for Malaria Research (ICEMR) surveys from Malawi in which blood samples of all individuals from selected households in Blantyre, Thyolo and Chikhwawa were tested for *Pf* parasitemia. We assessed household SEP and potential mediators – housing quality, food security, education status of household heads, and use of long-lasting Insecticide-treated nets (LLINs) and nutritional status. We conducted causal mediation analyses to assess the proportion of SEP effect that is attributed to each mediator and combination of mediators. The mediation analysis shows that during the rainy season, improved housing and educational attainment explained 39.4% and 17.0% of the SEP effect on *Pf* infection, respectively, and collectively 66.4%. In the dry season, housing, educational attainment, and LLIN usage collectively mediated 33.4% of SEP’s effect with individual contributions of 15.6%, 11.2%, and 3.8%, respectively. Nutrition also played a role, particularly for children, mediating 9.2% of SEP’s effect in the rainy season and 3.7% in the dry season. The study concluded that multifaceted interventions targeting housing, education, LLIN usage, and nutrition are vital to reducing socioeconomic disparities in *Pf* infection risk in the Southern region of Malawi.

**Supplementary Information:**

The online version contains supplementary material available at 10.1038/s41598-024-78512-1.

## Introduction

Malaria remains a leading public health problem in sub-Saharan Africa (SSA), accounting for over 95% of the global malaria cases and deaths^1^. In Malawi, malaria, caused by *Plasmodium falciparum (Pf)*infection, is responsible for approximately 40% of all outpatient visits and 36% of all hospital deaths^2^. The disease remains endemic in most parts of the country, with transmission occurring throughout the year although it peaks during the rainy season. The transmission also tends to increase with a decrease in altitude as lower altitudes are characterized by temperatures above 17 °C, which favour the breeding of and development of vectors and parasites^3,4^. *Pf*infection transmission is heterogeneous and may be influenced by a variety of factors including socioeconomic position, climate, ecology, vector behaviour, and use of malaria preventive interventions^5^.

Socioeconomic position (SEP), one of the key determinants of *Pf infection *risk, affects both the exposure to infection and the vulnerability of individuals and communities to the disease^6^. Evidence of socioeconomic inequities in relation to malaria in SSA has been well documented^7–10^, with prior research indicating higher odds of malaria among persons in low SEP compared to those in high SEP households^11,12^. For example, low SEP can also affect the living conditions that favour or limit the breeding and survival of Anopheles mosquitoes, the vectors of *Plasmodium *parasites^13,14^. Households with a lower SEP are more likely to have poorer housing quality (i.e., not fully sealed), which can increase exposure to mosquitoes. Further, greater presence of open water sources due to limited access to piped water, can increase the presence of larval habitats around their dwellings^15,16^.

Understanding the dynamics of how SEP influences *Pf infection* risk is complex and includes both direct and indirect effects. Previous studies have suggested that the effect of SEP on *Pf infection* is largely indirect^17,18^ and hence mediated by other proximal factors on the causal pathway, such as improved housing, educational attainment, nutrition, food security, access to health care, use of long-lasting treated nets (LLINs), and indoor residual spraying^17,19,20^. However, there is limited evidence to confirm and quantify their mediating role in the association between SEP and *Pf infection*in different settings^17,19,20^. Identifying the mediating pathways can inform more effective interventions against malaria after estimating how much of the effect can be eliminated by targeting these pathways^21,22^. At present, studies that examine the mediators of the association between SEP and *Pf infection*in SSA are limited^17,23^. The few studies that exist primarily employ traditional analytical methods. These methods often overlook the confounding factors that may affect the mediator-exposure relationship and fail to consider the collective effect of multiple mediators^7,24^. This study aims to address this gap by using a causal mediation analyses to examine how improved housing quality, educational attainment of household head, food security, nutrition and LLIN use mediate the association between SEP and *Pf infection* in the Southern region of Malawi. Our findings will provide new insights into the multifaceted pathways that link SEP and *Pf infection*, and inform more effective and equitable strategies towards achieving malaria elimination in Malawi.

## Methods

### Study setting

The Malawi International Center of Excellence for Malaria Research (ICEMR, U.S. N.I.H. U19AI089683) conducted cross-sectional surveys between 2010 and 2017 in Malawi - a country in southern part of Africa where malaria is endemic with seasonal variation and peaks during rainy seasons. Malaria in Malawi is mainly caused by *Plasmodium falciparum*, a parasite which is transmitted by several species of anopheles mosquitoes including *Anopheles gambiae*, *Anopheles arabiensis*, and *Anopheles funestus*^25^. The two ICEMR survey rounds in 2014 focused on three of Malawi’s 28 districts: Blantyre, Chikhwawa, and Thyolo. These districts are located in the south of the country, have a total population of over 2.5 million people and cover a total area of 8,569 km^26^. The malaria transmission patterns in the three districts are quite heterogeneous. Chikhwawa is a rural low-land district, which has more intensive transmission with a previous reported *Pf infection*prevalence of 20% in the dry and 32% in the rainy season^27^. Thyolo is a rural mountainous district with largely moderate transmission and a reported prevalence of 10% in dry and 15% in rainy seasons. In contrast, Blantyre is predominantly urban and is the second largest city in the country after Lilongwe with generally low levels of transmission with prevalences of 5% in the dry season and 9% in the rainy season^25^.

### Study design

The study’s design and sampling methodologies are elaborated upon in prior publications^27,28^. In summary, two cross-sectional surveys were conducted in 2014, corresponding with Malawi’s climatic seasons: one during the rainy season (April-May) and the other in the dry season (September-October). Each survey targeted a total sample size of at least 900 households, with each district contributing 300 households.

A two-stage sampling approach was employed. Initially, 10 enumeration areas (EAs) per district were selected randomly using probability proportionate to size. In Blantyre, only EAs within the city were sampled. In Thyolo, EAs bordering Chikhwawa or those at less than 500 m above sea level were excluded. Similarly, in Chikhwawa, EAs bordering Thyolo or EA at greater than 500 m above sea level were excluded. If a randomly selected EA met the exclusion criteria, it was replaced by another randomly selected EA. Next, 30 households within each EA were selected using compact segment sampling. Each selected EA was divided into segments of approximately 30 households and one segment was randomly selected. All households within the selected segment were visited, and the household head along with all consenting household members were invited to participate in the study^27^. For the dry season survey, the research teams revisited the same 30 EAs and primarily interviewed participants from households that were initially surveyed in the rainy season. It is important to note that while the same EAs were revisited, there was no specific tracking/linking of individual households or participants between the two survey rounds. This study has followed the guidelines for reporting mediation analysis studies^29^. The reporting checklist is available as Supplementary File 1.

### Data collection procedures

Detailed procedures were previously published^27^. Briefly, written informed consent for participation was obtained from the head of the household, each adult household member, and a parent/guardian for all minors. Assent was obtained from children 13–17 years old. Questionnaires based on the Malaria Indicator Survey^30^ were used to collect demographic data (district, age, gender, household size, educational attainment of household head), durable assets owned, LLIN ownership and use, and information regarding food security. Housing characteristics (roof type, floor type, wall type, house eaves, and windows) were observed. Fingerpick blood samples were taken from all participants for malaria PCR testing (whole collected and dried on filter paper) and from children aged 6 months to 15 years for haemoglobin testing (Hemocue^®^)^31^.

### Measurement and variables

The ***outcome*** was a PCR-confirmed *Plasmodium falciparum (Pf)* infection (which included asymptomatic or symptomatic individuals assessed during active case detection). DNA was extracted from filter papers and subsequently subjected to real-time PCR targeted to the *Pf*lactate dehydrogenase gene as previously described^27^. Real-time PCR allows for accurate, automated and real-time detection of target material, unlike conventional PCR. This enables faster acquisition of laboratory results, hence guiding infected participants to seek timely and appropriate management^32^. A positive PCR test result was indicative of *Pf infection* (coded 1), otherwise negative (coded 0).

The ***main exposure*** was household SEP, for which we constructed a wealth index using principal component analysis (PCA) of durable assets, such as ownership of radio, television, telephone, bicycle, house, electricity, and cooking fuel (clean vs. non-biomass), any income source (yes vs. no), as well as average number of persons per room (≤ 2 vs. > 2). We did not include variables on housing construction, as housing conceptually lies on the causal pathway between SEP and *Pf infection* and may have an independent direct effect on *Pf infection*risk and was hence considered as a mediator instead^6^. In our analysis, we first examined the descriptive statistics for the variables considered for PCA to assess missingness. Given that missingness was less than 0.1%, we proceeded with a complete case analysis. We used a tetrachoric correlation structure and opted not to rotate the principal components (PCs) since the rotated and non-rotated PCs yielded comparable results. The first PC, with an eigenvalue of 4.06, accounted for 45% of the total variance and was used to derive the wealth index scores. These scores were then divided into three categories—lower, medium, and high—creating a new SEP variable. The categorization followed a 40:40:20 ratio to allow for easier interpretation of the results^33,34^.

***Mediators*** for *Pf infection* and SEP included housing quality, highest educational attainment of the household head, use of LLINs, food security, and nutrition (available only for participants aged 6 months to 15 years).

- **Housing quality** served as a mediator in the association between SEP and *Pf infection*. Specifically, housing quality was categorized into two types: modern and traditional. Modern houses were those that met at least three out of the following four criteria: finished materials for the roof, floor, walls, and closed eaves. In contrast, traditional houses met less than 3 of these criteria (0 to 2 of the conditions). In line with the definitions of the Demographic and Health Surveys (DHS)^32^, finished walls refer to those made of materials, such as cement, bricks, cement blocks, covered adobe, plastered wall, stone with lime and wood planks; while rudimentary walls refer to those made of materials, such as cane, palm, bamboo or stone with mud, and plywood. Finished roofs refer to those made of materials, such as metal sheets, wood, cement and roofing shingles, while natural or rudimentary roofs are made of materials, such as thatch, stick and mud, plastic sheet, bamboo, and wood planks. Finished floors refer to those made of materials, such as parquet or polished wood, vinyl or asphalt strips, ceramic tiles, cement, and carpet, while natural or rudimentary floors are made of materials, such as earth, sand, dung, wood planks, palm, and bamboo.
- **Highest educational attainment**: The highest educational attainment of the household head was categorized as follows: ‘none’, ‘primary’, and ‘secondary or higher’. These categories were coded as 0, 1, and 2, respectively.
- **Food security** was assessed based on three questions posed to the household head: In the past four weeks, how often did you or a household member (1) worry about not having enough food, (2) eat smaller meal sizes than would normally be considered enough, and (3) eat fewer meals in a day because of lack of food. The response categories to these questions were none, rarely, sometimes or often. We created a binary indicator variable in which a household was considered food secure (coded 1) if the frequency of none of the three scenarios was present, otherwise categorized as food insecure (code 0).
- **Use of long-lasting insecticide-treated nets (LLINs)**: This is a mediator at the individual level and was based on the question “whether a person (participant) slept under LLINs the previous night”. Affirmative responses were coded 1, otherwise coded 0. In addition, we assessed LLIN universal coverage, which is defined as having a maximum of two people per LLIN on average in a household, but we used reported LLIN use in our mediation models^35^.
- **Nutrition status**: In this study, nutritional status was assessed among children aged 6 months to 15 years, using anaemia status as a proxy marker. Haemoglobin concentrations of ≥ 11.0 g/dl for children 6–59 months, 11.5 g/dl for children 5–11 years, and ≥ 12.0 g/dl for children 12–15 years were considered indicative of being free from anaemia. Children meeting these criteria were coded as ‘1’, and those not meeting them were coded as ‘0’^36^. The mediating role of nutrition status was determined only for children (6 months to 15 years). It is imperative to note that while anaemia can reflect suboptimal nutritional status, it may also arise as a consequence of malaria. In areas endemic for malaria, the disease itself can precipitate anaemia through hemolysis and other pathophysiological processes. Consequently, the presence of anaemia might be a result of, rather than a precursor to, malaria infection. This consideration is crucial when interpreting the mediation analysis findings as they may not be accurate.

**Covariates** for this study included the age of participants (< 5 years, 5–15 years and 16 + years), geographical location (Blantyre, Chikhwawa, and Thyolo), sex (male and female), and household size as discrete variables. Missingness for outcome, mediators, and key covariates is shown in Supplementary File 2.

### Statistical analysis

We used descriptive statistics to summarize the baseline characteristics of households and participants in the positive *Pf infection* and -negative groups and report p-values adjusted for multiple testing. We reported numerical data as means and standard deviations and categorical data as frequencies and percentages. We used two separate regression models to assess the association between SEP and malaria as follows: For each of the seasons (rainy and dry), we ran multivariable mixed-effects modified Poisson regression models with household as a random intercept since participants from the same household are correlated. We adjusted for age, sex, household size and geographical location, and reported prevalence ratios (PRs) and 95% confidence intervals (CIs). We subsequently conducted causal mediation analyses for the association between SEP and *Pf infection using the CMaverse package*^37^. We examined the proportion mediated by improved housing quality, LLIN use, food security, highest educational attainment of the household head and anaemia free status (only among children ≥ 6 and ≤ 15 years). A Directed Acyclic Graph (DAG) for this association is depicted in Supplementary File 3. We used regression-based approaches allowing for potential exposure-mediator interactions (where possible) to estimate the total effect (TE), total natural indirect effect (TNIE), and total natural direct effect (TNDE). The TNIE was the effect of SEP on *Pf infection* explained by its association with the mediators individually and in combination. The TNDE was the effect of SEP on *Pf infection* independent of the mediator. We estimated the proportion of the association mediated by the mediator as TNIE/ [TNDE + TNIE].

We used non-parametric bootstrapping with multiple imputation to handle any missing data for some mediators (such as LLINs use (0.1%), educational attainment of household heads (0.6%), anaemia free status among those aged between 6 months and 15 years, (2.52%) and covariates, age (0.2%) and to obtain robust PRs with 95% CIs. The counterfactual assumptions included (*i*) no unmeasured confounding of the treatment-outcome relationship (*ii*) no unmeasured confounding of mediator-outcome relationship iii) no unmeasured confounding of the treatment-mediator relationship. We assumed a temporal sequence between exposure and outcomes and also conducted sensitivity analyses to assess the robustness of estimates to unmeasured confounding and reported the mediational E-values and corresponding 95% CIs closest to null. Baron and Kenny’s criteria for identifying a potential mediator are provided in Supplementary File 4. Additional analyses for mediation effects based on different age groups and seasons are provided in Supplementary File 5. All analyses were run using R software.

## Ethical considerations

The study obtained ethical approval from the independent Institutional Review Boards (IRBs) of the University of Malawi College of Medicine, the University of Maryland, Baltimore, and Michigan State University. The studies were performed in accordance with relevant guidelines including the Declaration of Helsinki. Informed consent was obtained from all study participants and/or their legal guardians.

## Results

### Individual and household characteristics by *Pf* infection status

During the study, 3,003 individuals participated in the rainy season survey, while 3,253 took part in the dry season. The majority of participants were female (61.3% in the rainy season and 60.9% in the dry season).

The prevalence of *Plasmodium falciparum (Pf)* infection differed between seasons; it was 19.7% (95% Confidence Interval, CI: 18.4 – 21.3%) in the rainy season, which decreased to 12.9% (95% CI: 11.8 – 14.1%) in the dry season. Reports of fever within the last 48 h were 7.5% in the rainy season, dropping to 3.8% in the dry season. Notably, 80.5% of participants exhibited no symptoms during the rainy season, with a slight increase to 83.8% in the dry season. Table 1 summarizes the individual and household characteristics by *Pf* infection status across both seasons.

**Table 1:** Individual and household characteristics by *pf infection* status and by season.

Characteristic	Rainy season (April /May 2014) (*N* = 3,003)				Dry season (Sept/Oct 2014) (*N* = 3,253)			
	*N* = 3,003^1^	*Pf* infection, *N* = 594^1^	No *Pf* infection, *N* = 2,409^1^	p-value*	*N* = 3,253^1^	*Pf* infection, *N* = 421^1^	No *Pf* infection, *N* = 2,832^1^	p-value*
District				< 0.001				< 0.001
Blantyre	998 (33.2%)	85 (8.5%)	913 (91.5%)		1,050 (32.3%)	56 (5.3%)	994 (94.7%)	
Chikhwawa	1,043 (34.7%)	361 (34.6%)	682 (65.4%)		1,173 (36.1%)	261 (22.3%)	912 (77.7%)	
Thyolo	962 (32.0%)	148 (15.4%)	814 (84.6%)		1,030 (31.7%)	104 (10.1%)	926 (89.9%)	
Age of participant				< 0.001				< 0.001
16+	1,339 (44.7%)	232 (17.3%)	1,107 (82.7%)		1,379 (42.5%)	125 (9.1%)	1,254 (90.9%)	
5–15	1,106 (36.9%)	268 (24.2%)	838 (75.8%)		1,272 (39.2%)	235 (18.5%)	1,037 (81.5%)	
under 5	552 (18.4%)	94 (17.0%)	458 (83.0%)		595 (18.3%)	60 (10.1%)	535 (89.9%)	
Sex of the participant				0.326				0.011
Female	1,835 (61.2%)	353 (19.2%)	1,482 (80.8%)		1,977 (60.9%)	231 (11.7%)	1,746 (88.3%)	
Male	1,164 (38.8%)	241 (20.7%)	923 (79.3%)		1,271 (39.1%)	189 (14.9%)	1,082 (85.1%)	
Household head education attainment				< 0.001				< 0.001
None	605 (20.2%)	172 (28.4%)	433 (71.6%)		601 (18.6%)	109 (18.1%)	492 (81.9%)	
Primary	1,668 (55.8%)	332 (19.9%)	1,336 (80.1%)		1,896 (58.7%)	267 (14.1%)	1,629 (85.9%)	
Secondary or higher	715 (23.9%)	85 (11.9%)	630 (88.1%)		731 (22.6%)	41 (5.6%)	690 (94.4%)	
Household size	4.6 (1.8)	4.8 (1.6)	4.6 (1.8)	0.015	4.8 (1.7)	4.9 (1.7)	4.7 (1.7)	0.040
Fever in the last 48 h								
No	2779 (92.5%)	518 (18.6%)	2261 (81.4%)	< 0.001	3129 (96.2%)	398 (12.7%)	2731 (87.3%)	0.067
Yes	224 (7.5%)	76 (33.9%)	148 (66.1%)		124 (3.8%)	23 (18.5%)	101 (81.5%)	
Socioeconomic strata				< 0.001				< 0.001
Lower	1,223 (40.7%)	311 (25.4%)	912 (74.6%)		1,177 (36.2%)	200 (17.0%)	977 (83.0%)	
Middle	1,194 (39.8%)	218 (18.3%)	976 (81.7%)		1,455 (44.7%)	195 (13.4%)	1,260 (86.6%)	
High	586 (19.5%)	65 (11.1%)	521 (88.9%)		621 (19.1%)	26 (4.2%)	595 (95.8%)	
Housing quality				< 0.001				< 0.001
Low	1,364 (45.4%)	379 (27.8%)	985 (72.2%)		1,507 (46.3%)	274 (18.2%)	1,233 (81.8%)	
High	1,639 (54.6%)	215 (13.1%)	1,424 (86.9%)		1,746 (53.7%)	147 (8.4%)	1,599 (91.6%)	
Food security				0.015				0.391
Insecure	493 (16.4%)	118 (23.9%)	375 (76.1%)		403 (12.6%)	57 (14.1%)	346 (85.9%)	
Secure	2,509 (83.6%)	476 (19.0%)	2,033 (81.0%)		2,798 (87.4%)	353 (12.6%)	2,445 (87.4%)	
Household owns LLIN(s)				0.132				< 0.001
No	487 (16.2%)	109 (22.4%)	378 (77.6%)		877 (27.0%)	151 (17.2%)	726 (82.8%)	
Yes	2,516 (83.8%)	485 (19.3%)	2,031 (80.7%)		2,376 (73.0%)	270 (11.4%)	2,106 (88.6%)	
At most 2 persons share a LLIN				< 0.001				< 0.001
No	2,201 (73.3%)	475(21.6%)	1,726 (78.4%)		2584 (79.4%)	365 (14.1%)	2,219 (85.9%)	
Yes	802 (26.7%)	119 (14.8%)	683 (85.2%)		669 (20.6%)	56 (8.4%)	613 (91.6%)	
Slept in a LLIN the previous night				0.132				< 0.001
No	1,056 (35.2%)	225 (21.3%)	831 (78.7%)		1,863 (57.3%)	302 (16.2%)	1,561 (83.8%)	
Yes	1,947 (64.8%)	369 (19.0%)	1,578 (81.0%)		1,390 (42.7%)	119 (8.6%)	1,271 (91.4%)	
Nutrition (Anemia status)^3^				< 0.001				< 0.001
Anaemia	876 (54.5%)	237 (27.1%)	639 (72.9%)		921 (50.0%)	175 (19.0%)	746 (81.0%)	
No Anaemia	730 (45.5%)	104 (14.2%)	626 (85.8%)		920 (50.0%)	114 (12.4%)	806 (87.6%)	

^1^n (%); Mean (SD).

^2^Pearson’s Chi-squared test; Welch Two Sample t-test; *p-values corrected (Benjamini & Hochberg correction for multiple testing).

^3^Assessed only among participants aged 15 years and younger.

Note: 23.9% of participants had secondary or higher education in the rainy season (2.5% tertiary), compared to 22.6% in the dry season (1.2% tertiary).

### Association between SEP and *Pf infection*during rainy and dry seasons

We assessed the association between SEP of the households and *Pf* infection in the rainy and dry seasons. Our main findings include that there was no association between household wealth and *Pf* infection risk in the rainy season (PR = 0.83, 95% CI = 0.52–1.33) and the dry season (PR = 0.57, 95% CI = 0.32–1.03). Having secondary or higher education and better housing were protective in both seasons while LLIN use appeared to be protective in the dry season (PR = 0.52, 95% CI = 0.39–0.71) (Table 2).

**Table 2:** Association between SEP and *Pf infection* during the rainy and dry seasons.

Characteristic	Rainy season (*N* = 3,003)			Dry season (*N* = 3,253)		
	PR^1^	95% CI^1^	p-value*	PR^1^	95% CI^1^	p-value*
Sex of participants						
Male	—	—		—	—	
Female	0.98	0.79–1.21	0.850	0.91	0.72–1.16	0.508
Age categories						
Under 5	—	—		—	—	
5–15	1.61	1.25–2.07	< 0.001	1.84	1.35–2.51	< 0.001
16+	1.81	0.25–13.3	0.644	2.31	0.30–18.1	0.508
Socioeconomic strata						
Lower	—	—		—	—	
Middle	1.07	0.83–1.39	0.644	1.18	0.88–1.59	0.349
High	0.83	0.52–1.33	0.644	0.57	0.32–1.03	0.128
Educational attainment of household head						
None	—	—		—	—	
Primary	0.73	0.56–0.95	0.038	0.81	0.59–1.11	0.291
Secondary or higher	0.53	0.36–0.78	0.004	0.48	0.28–0.80	0.017
Household size	1.11	1.03–1.19	0.014	1.06	0.97–1.15	0.291
Food Security	0.89	0.67–1.17	0.644	0.99	0.67–1.46	0.968
Slept in a LLIN the previous night	0.93	0.74–1.16	0.644	0.52	0.39–0.71	< 0.001
Housing quality (modern housing)	0.54	0.41–0.71	< 0.001	0.69	0.51–0.93	0.035
Nutrition (Anemia status)	0.54	0.41–0.71	< 0.001	0.64	0.49–0.84	0.005

^1^PR = Prevalence Ratio, CI = Confidence Interval, * Benjamini & Hochberg correction for multiple testing.

## Mediation analysis

### Mediators on the pathway between SEP and *Pf infection* in the rainy season

In the rainy season, the single mediator analyses showed that housing quality and having secondary or higher education partially mediated 39.4% and 17.0% of the effect of SEP on *Pf infection*, respectively. The multiple mediator analyses showed that the combined effect of both mediators explained 66.4% of the effect of SEP on *Pf infection*. The mediated effects through housing were consistent across age groups (Supplementary File 5). In the subset of participants aged 6 months to 15 years, nutrition status mediated 9.2% of the effect of SEP on *Pf infection* in the rainy season.

**Fig. 1:**
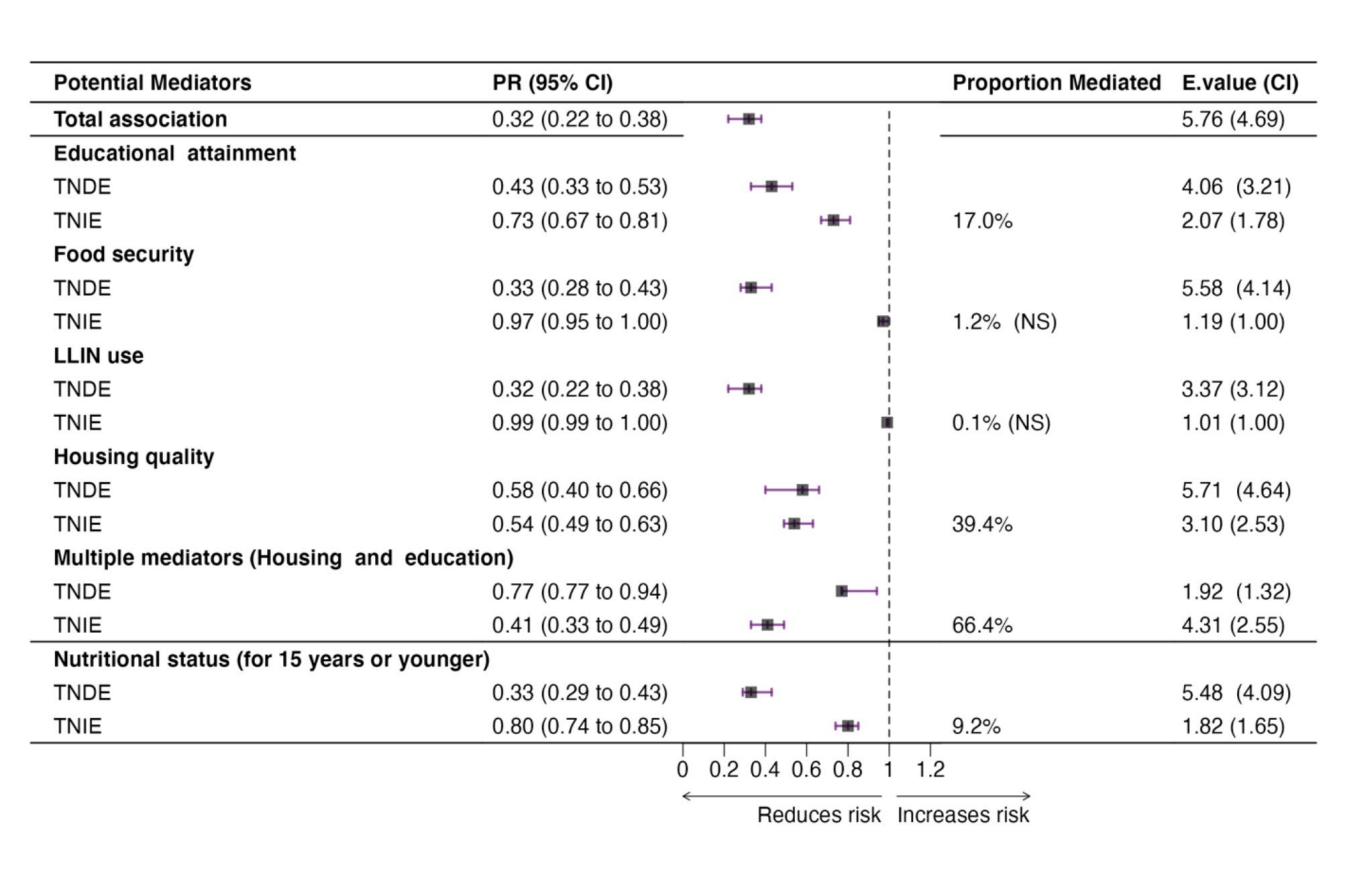
Mediation analysis for the association between SEP and *Pf infection* in the rainy season. *All mediation models adjusted for age, sex and household size; multiple mediators mean a combination of mediators including potential interactions. Abbreviations: PR: Prevalence Ratio, TNDE: Total Natural Direct Effect, TNIE: Total Natural Indirect Effect. CI associated with the E-value represents the E-value of the confidence interval of the indirect effect closest to null.

Although indirect effects through multiple mediators were more robust to unmeasured confounding as seen by E-values, the mediated (indirect) effects through individual mediators such as housing and educational attainment were less robust. This suggests that a relatively small confounding effect could potentially explain the observed mediated estimates through individual mediators. The E-value which assesses the impact of unmeasured confounding, has values as low as 1 and +∝. Larger E-values indicate a stronger association that is less likely to be nullified by unmeasured confounders, while lower E-values signal vulnerability to such confounding (especially when close to 1) (Fig. 1).

#### Mediators on the pathway between SEP and *Pf infection* during the dry season

In the dry season, better housing quality, secondary school or higher education, and LLIN use partially mediated 15.6%, 11.3%, and 3.8% of the effect of SEP on *Pf infection*, respectively. Multiple mediator analyses show that these three jointly mediated 33.4% of the effect of SEP. Among children aged 15 years and younger, nutrition status mediated 3.7% of the effect of SEP on *Pf infection*.

**Fig. 2:**
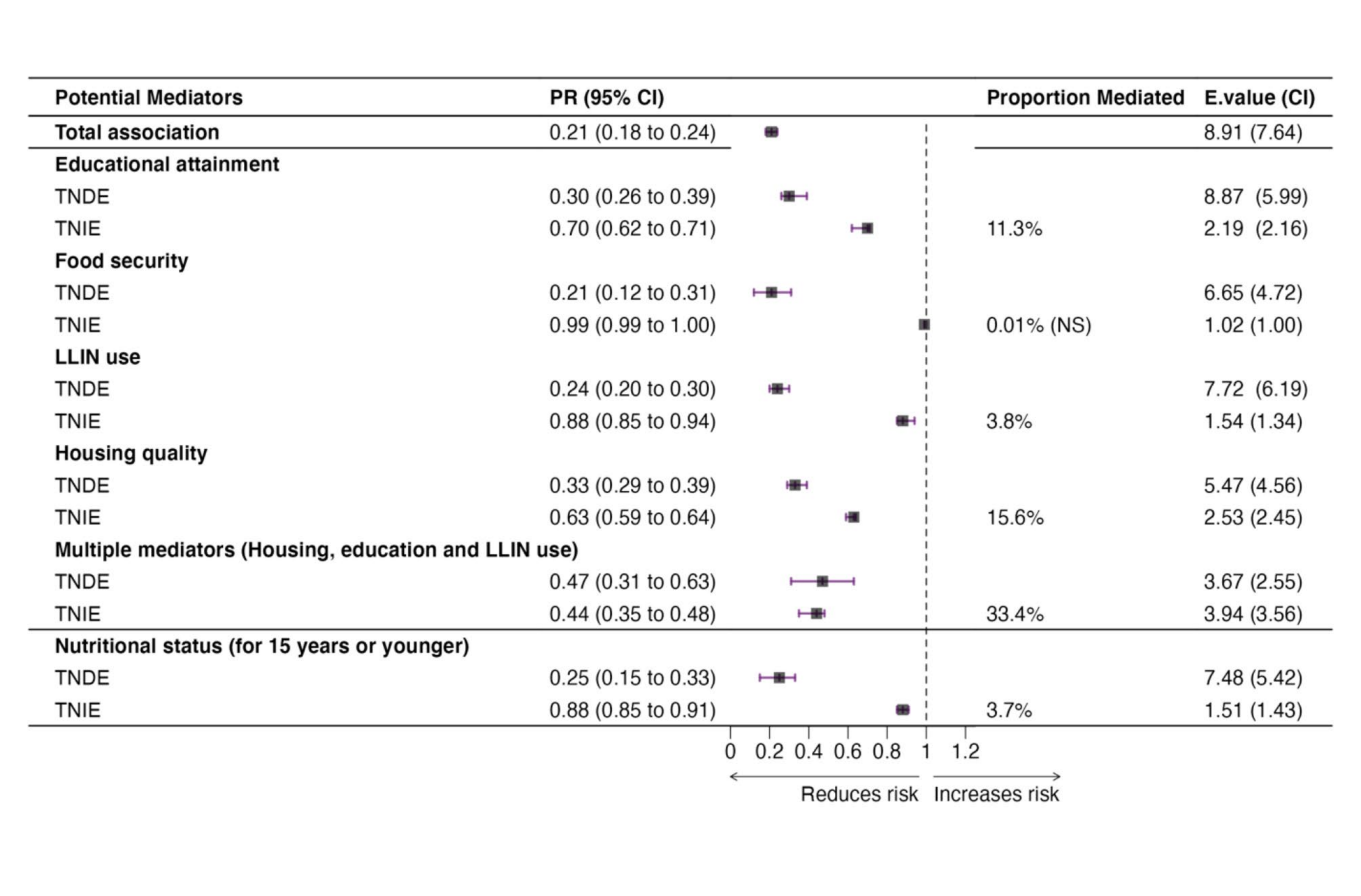
Mediation analysis for the association between SEP and *Pf infection* during the dry season. *All mediation models adjusted for age, sex and household size; multiple mediators mean a combination of mediators including potential interactions. Abbreviations: PR: Prevalence Ratio, TNDE: Total Natural Direct Effect, TNIE: Total Natural Indirect Effect. CI associated with the E-value represents the E-value of the confidence interval of the indirect effect closest to null.

The TNIEs from individual mediator analyses were again less robust to unmeasured confounding while that from multiple mediator analyses was moderately robust (Fig. 2).

## Discussion

In this study, we examined the prevalence of *Pf* infection in the general population in Southern region of Malawi and investigated/examined the direct and indirect (mediated) effects of SEP on *Pf infection* through potential mediators- housing quality, food security, LLIN use, educational attainment of household heads and nutrition status (haemoglobin) during dry and rainy seasons. The finding revealed that 19.7% of the participants had *Pf* infection during the rainy season, while 12.9% had the *Pf* infection during the dry season, underscoring the role of seasonality in *Pf*infection transmission patterns. Although these prevalence rates are high, they are comparatively lower than 37%^38^and 35.4%^39^reported in recent studies from Malawi. However, during the rainy season, the lowland Chikhwawa district had a comparable prevalence of 34.6%. The aforementioned studies focused solely on children under five and used different sampling approaches which may explain the variation in prevalence estimates. Additionally, there is well-documented significant heterogeneity in malaria transmission rates across different regions and districts in Malawi^25^.

We found that higher SEP was associated with a lower probability of having *Pf* infection. This association was partially mediated by housing quality during both rainy and dry seasons with a more pronounced effect in the rainy season. The fact that higher SEP was strongly associated with improved housing, and improved housing was protective against *Pf infection*fits Baron and Kennys’ main criteria for a potential mediator^40^. Improved housing may prevent the entrance of mosquito vectors and thereby reduce the mosquito biting rate, especially at night/evenings, which in turn reduces the risk of *Pf*infection^41^. Since most transmission occurs at night^42^, the dwelling can be a risky place and needs to be well constructed and screened to prevent mosquito entry. Furthermore, although our study found a protective mediated effect of SEP through housing, it is important to note the shifting vector behaviour in response to widespread LLIN use over the last few years with more biting now occurring in the evening and morning and this may render housing less effective^43^.

We also found that reported LLIN use explained 3.8% of the effect of SEP on *Pf infection* in the dry season, a pattern not replicated in the rainy season. This underscores the protective effectiveness of LLINs against *Pf infection*and the influence of SEP on their usage as previously highlighted^18,44,45^. In Malawi, universal bed net coverage has not yet been achieved, because insufficient amounts of LLINs are distributed for free^46^. The absence of a mediated effect during the rainy season could be attributed to increased mosquito exposure during outdoor activities and a higher indoor mosquito population, which means people have a higher chance of getting bitten before they use the LLINs whilst sleeping in the evenings. This negates the protective effects of LLIN to some extent and necessitates the use of additional control measures.

Among children aged 15 years and younger, we found that improved nutritional status mediated 9.2% of the effect of SEP on the risk of *Pf infection* during the rainy season, and 3.7% during the dry season. This mediating effect was evident across both under-5 and SAC in the rainy season but was limited to SAC in the dry season. Previous research has reported mixed findings regarding the associations between nutritional status and *Pf infection*^47,48^, yet there is growing consensus that malnutrition may increase susceptibility to *Pf*infection^49^. Traditional measures of nutritional status, such as stunting or wasting, have been used in these studies, but they fail to capture micronutrient deficiencies, that can occur even in children who are neither stunted nor wasted. Our study employed haemoglobin levels as a proxy for nutritional status, given its strong correlation with various indicators of malnutrition across all age groups^50^. However, it is crucial to interpret this association with caution. The cross-sectional nature presents difficulties in establishing a clear temporal sequence, especially in light of the known bidirectional relationship between anaemia and *Pf*infection especially in endemic settings such as Malawi^51^. Despite these complexities, the link between malnutrition and compromised immune function—which heightens the risk of infections such as Pf—cannot be overlooked but rather needs to be investigated in a truly longitudinal design where we can accurately determine temporarity. Nonetheless, improving nutritional status may be vital to mitigate some of the poverty-related impacts on the incidence of *Pf infection*by enhancing immune defences^49^.

Education has been shown to have a positive impact on a whole range of health-related outcomes. In this study, we found that having household heads educated to tertiary level partially mediated the effect of SEP on the risk of *Pf infection* among household members. This was also confirmed in seasonal and aged-based sub-group analyses – except for adults in the dry season. We hypothesize that higher SEP enables high educational attainment and when household heads are well-educated, then they are more likely to know about and use preventive measures (Supplementary File 6), such as using LLINs, clearing breeding grounds, proactive acquisition of insect sprays, which consequently reduces exposure to mosquito vectors and hence *Pf infection*risk^52^. Moreover, higher education also comes with more financial power to procure some of these preventive measures but may also facilitate earlier symptom recognition and timely treatment seeking^53^. This shows that as part of the long-term efforts to eradicate malaria, improving education attainment is an important socio-structural intervention that should be promoted.

Our findings indicate that employing a combination of mediators—improved housing and education, during the rainy season, and the same factors with LLIN usage in the dry season—had a synergistic effect on reducing *Pf infection* risk. Targeting multiple mediators appears to be more effective than single-mediator strategies and findings were more robust to unmeasured confounding. Notably, the impact of these mediators is stronger during the rainy season, a period of heightened malaria transmission, underscoring their critical importance. In the rainy season, the combined effect of these mediators is particularly pronounced in adults but also children under 5 who represent the age group with the highest malaria-related mortality rates. The relatively lower indirect effect among the SAC suggests the existence of alternative pathways through which SEP influences *Pf infection*risk in this subgroup, which merits further investigation. Conversely, in the dry season, the proportion of the mediated effect of SEP is considerably lower. These may be due to decreased mosquito populations, a corresponding lower transmission rate, and possibly lower utilization of LLINs in the dry as compared to during the rainy season. Moreover, evidence shows that a higher proportion of dry season pf infections are chronic as opposed to incident, acute infections. Thus, they may be less influenced by exposure-related mediators and more related to other factors such as immunity, and age^54^ These findings suggest the need for constant adaptation of preventive strategies in different seasons. Additionally, for individuals aged between 6 months to 15 years, the inclusion of better nutritional status with other mediators (housing, educational attainment and LLIN use (dry season)) further enhances the mediated protective effect against *Pf infection*, particularly in the rainy season, again highlighting the importance of season-specific intervention strategies. The results underscore the need for integrated malaria control measures that address multiple risk factors simultaneously.

This is one of the first studies to consider several socio-structural mediators of the association between SEP and *Pf infection*, individually and jointly, adding to the body of knowledge on the relationship between wealth/poverty and *Pf infection*. Our study contributes to the malaria control efforts by providing more comprehensive results from a counterfactual perspective. Our study also utilized PCR-based *Pf*test results, ensuring high sensitivity, and specificity, and the ability to detect submicroscopic malaria unlike microscopy or rapid diagnostic tests used in previous studies^55^. Limitations of this study include the inability to make causal claims due to the cross-sectional design and challenges around temporality, however, most of the exposures and mediators are structural and long-term (except for indicators of nutritional status), so we can be reasonably confident that they preceded the occurrence of *Pf* infection. Second, while the direct effect of SEP was robust to unmeasured confounding, the indirect/mediated effect through the individual mediators housing and LLIN use was less robust, which indicates that a confounding variable with a 2-fold increase in risk on a risk ratio scale would effectively sway the observed mediated effect to the null. However, this finding is still important in guiding the design of houses such as closing eaves or closing all possible entry points of mosquitoes inside the indoor spaces. Similarly, even a modest mediated effect through LLIN use suggests that improving universal LLIN coverage and use could help reduce *Pf infection* risk, highlighting the critical need to enhance distribution efforts. The third limitation is that the nature of the design violates the stringent identifiability assumptions required for causal interpretation, however, sensitivity analysis for unmeasured confounding was conducted to guide the causal interpretation. Lastly, the potential measurement errors in some of the mediators, such as food security and LLIN use (self-reporting bias) may introduce bias and affect the robustness of the findings, however, these results still provide an important source of hypotheses for future well-designed longitudinal studies.

## Conclusions

Improvements in housing quality, education, LLIN coverage and nutritional status have been highlighted as mediators and these warrant further investigation of their role in the association between poverty and *Pf infection* in longitudinal designs and settings. More potential mediators should be assessed to understand the complex pathways between poverty and *Pf infection* in different settings to be able to guide appropriate targeted structural interventions that can ensure sustained malaria control.

## Electronic supplementary material

Below is the link to the electronic supplementary material.

Supplementary Material 1

Supplementary Material 2

Supplementary Material 3

Supplementary Material 4

Supplementary Material 5

Supplementary Material 6

## Data Availability

Data are available upon request from corresponding author because the database contains personal identifiers. Any requests for the data will be reviewed by the relevant institutional review boards.

## Abbreviations

CI: Confidence Interval
DAG: Directed Acyclic Graph (DAG)
DHS: Demographic and Health Surveys
ICEMR: International Center of Excellence for Malaria Research
LLINs: Long-lasting insecticide-treated nets
PC: Principal Component
PCA: Principal Component Analysis
PCR: Polymerase Chain Reaction
*Pf*: *Plasmodium falciparum*
PR: Prevalence Ratio
SAC: School Age Children
SEP: Socioeconomic Position
SSA: Sub-Saharan Africa
TE: Total effect
TNIE: Total natural indirect effect
TNIE: Total natural direct effect
