# Supplementary material for: Socioeconomic disparities in Plasmodium falciparum infection risk in Southern Malawi: mediation analyses
Source: Sci Rep. 2024 Nov 8;14:27290. doi: 10.1038/s41598-024-78512-1 (PMC11549479; doi:10.1038/s41598-024-78512-1)
Supplement: Supplementary file 2 — Supplementary Material 2 [file 41598_2024_78512_MOESM2_ESM.docx]

**Additional file 2: Missingness on key variables**

*Overall: During dry (0) and rainy seasons (1) for all participants*

*
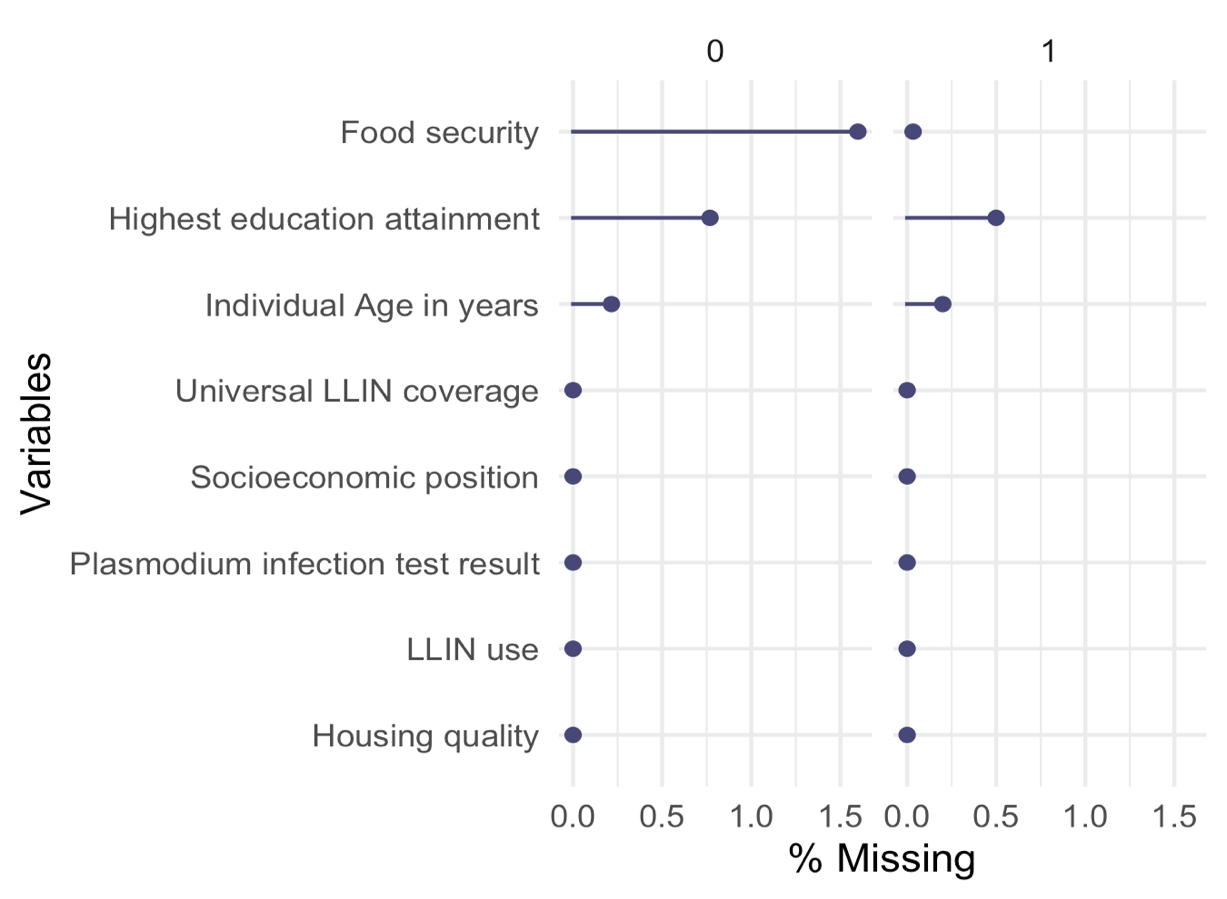
*

*Among participants aged between 6 months to 15 years*

*
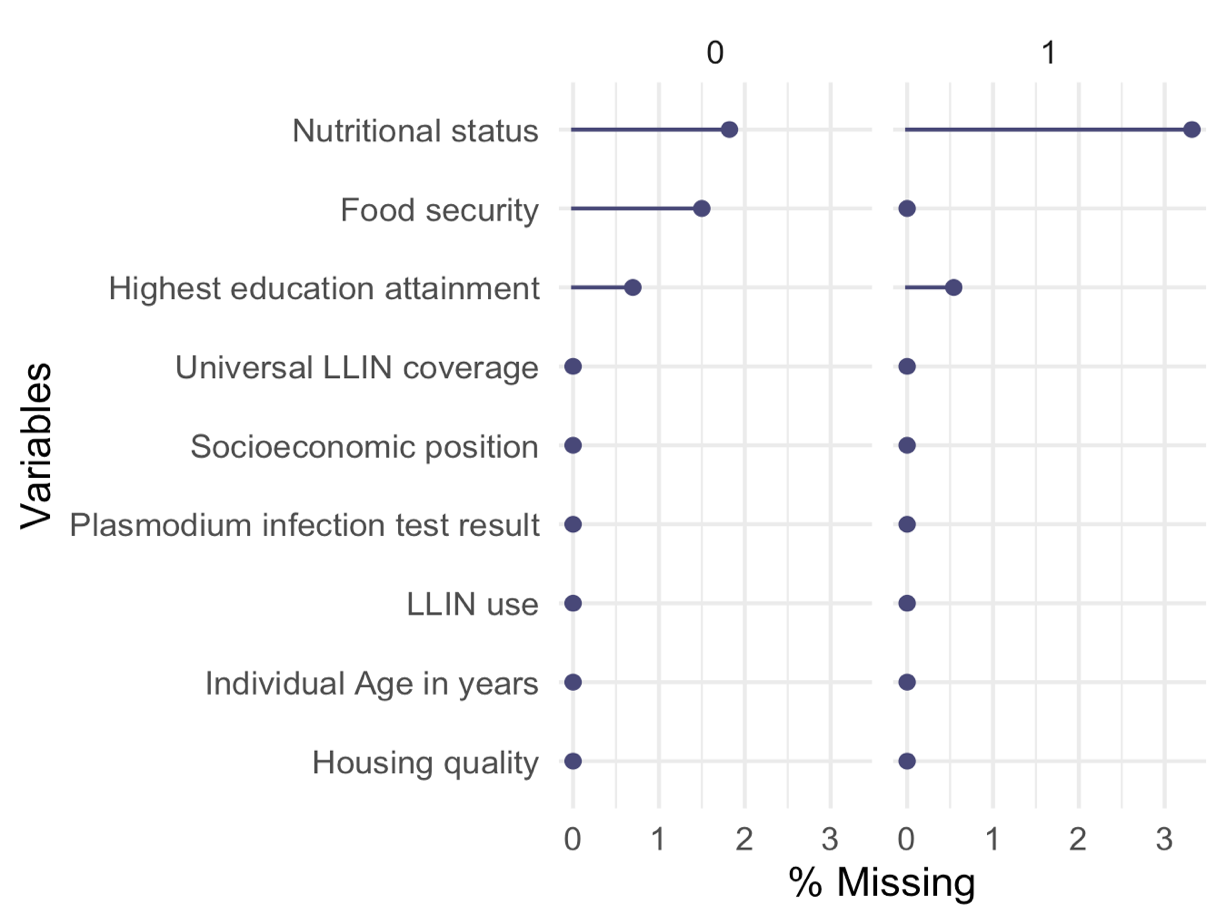
*

*Note: Based on the findings, missingness was very low (highest being approximately 3%); no definite missing pattern and appears to be missing at random (MAR), hence multiple imputation was considered appropriate in models*
