# Supplementary material for: Socioeconomic disparities in Plasmodium falciparum infection risk in Southern Malawi: mediation analyses
Source: Sci Rep. 2024 Nov 8;14:27290. doi: 10.1038/s41598-024-78512-1 (PMC11549479; doi:10.1038/s41598-024-78512-1)
Supplement: Supplementary file 3 — Supplementary Material 3 [file 41598_2024_78512_MOESM3_ESM.docx]

**Additional file 3: Directed acyclic graphs (DAG) for the study**

1. **DAG for the association between SEP and *Pf infection***


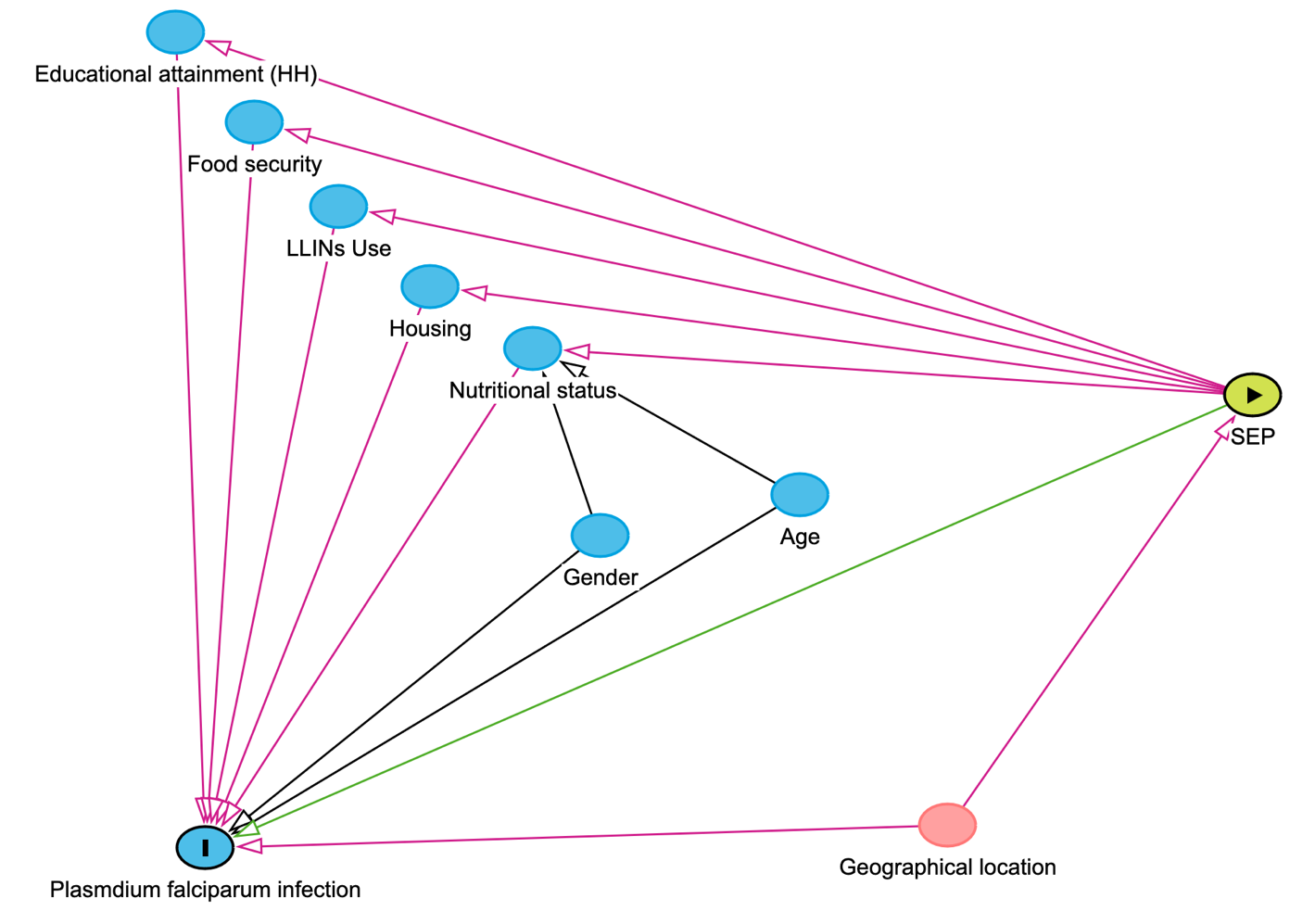


Figure 1. DAG for the association between SEP and Pf infection

Legend / notes:

| *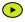* | *Exposure: Household SEP (SEP)* |
| --- | --- |
| *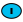* | *Outcome: Plasmodium falciparum infection* |
| *Mediators:* |  |
|  | - *Nutritional status,* - *Food security,* - *Housing quality,* - *LLIN use,* - *Education attainment* |
| *Covariates:* |  |
|  | - *Location* - *Age,* - *Gender* |
