# Supplementary material for: Socioeconomic disparities in Plasmodium falciparum infection risk in Southern Malawi: mediation analyses
Source: Sci Rep. 2024 Nov 8;14:27290. doi: 10.1038/s41598-024-78512-1 (PMC11549479; doi:10.1038/s41598-024-78512-1)
Supplement: Supplementary file 4 — Supplementary Material 4 [file 41598_2024_78512_MOESM4_ESM.docx]

**Additional file 4. Criteria for identifying mediator (The Baron and Kenny Approach, 1986)**

The causal diagram in Figure 2.1 captures the conceptualization of the role of a mediator variable. In this simple diagram, A represents an exposure variable, M denotes the mediating factor (mediator) and the outcome is denoted by Y.

M

Y

A

**Baron and Kenny suggested four criteria for identifying a mediator but only two are generally accepted as correct**

1. **A change in levels of the exposure variable significantly affects the changes in the mediator (i.e., Path *from A to M)***

In our study, SEP (change from poorer to richer) should affect changes in the mediator, the unadjusted estimates for the effect of SEP on mediators is shown below

**Table 1a. Unadjusted associations between SEP and the mediators**

|  | **Rainy season (N = 3,003)** | | **Dry season (N = 3,253)** | |
| --- | --- | --- | --- | --- |
| Mediators | Prevalence Ratio (PR), 95% Confidence interval (CI) | p-value^2^ | Prevalence Ratio (PR), 95% Confidence interval (CI) | p-value^2^ |
| **Housing quality**  (1=High, 0 = low) | 3.61 (3.45 – 4.13) | <0.001 | 3.49 (3.05 – 4.00) | <0.001 |
| **Food security**  (1= More secure, 0= less secure) | 1.18 (1.06 – 1.31) | 0.002 | 1.16 (1.04 – 1.28) | 0.006 |
| **LLIN coverage** (≤2 persons per net) | 1.88 (1.57 – 2.25) | <0.001 | 3.47 (2.83 – 4.27) | <0.001 |
| **LLIN use** (sleeping under treated net previous night) | 1.10 (0.97 – 1.24) | 0.145 | 1.91 (1.66 – 2.20) | < 0.001 |
| **Educational attainment**  (Post primary, primary, None) | 17.4 (13.9 – 21.8) | <0.001 | 17.3 (13.9 – 21.6) | < 0.001 |
| **Nutritional status**^1^  (1= No Anaemia, 0 =Anaemia) | 1.92 (1.59 – 2.32) | < 0.001 | 1.43 (1.20 – 1.71) | <0.001 |

^1^ Assessed among 6 months to 15-year-olds (Rainy season; N = 1606, dry season; N =1,841), ^2^p-values are corrected for multiple testing (Benjamin-Hochberg); In actual mediation analysis, we used LLIN use and not LLIN coverage.

1. **There is a significant relationship between the mediator and the outcome (i.e., Path from M to Y)**

We assessed crude associations between mediators and the outcome and presented the prevalence ratios and 95%CIs

**Table 1b.** **Unadjusted associations between each mediator and *Pf infection***

|  | **Rainy season (N = 3,003)** | | **Dry season (N = 3,253)** | |
| --- | --- | --- | --- | --- |
| Mediators | Prevalence Ratio (PR), 95% Confidence interval (CI) | p-value | Prevalence Ratio (PR), 95% Confidence interval (CI) | p-value |
| **Housing quality**  (1=High, 0 = low) | 0.47 (0.40 – 0.56) | <0.001 | 0.46 (0.38 – 0.56) | <0.001 |
| **Food security**  (1= More secure, 0= less secure) | 0.79 (0.65 – 0.97) | 0.024 | 0.89 (0.68 – 1.19) | 0.423 |
| **LLIN coverage** (≤2 persons per net) | 0.69 (0.56 – 0.85) | < 0.001 | 0.59 (0.44 – 0.78) | < 0.001 |
| **LLIN use** (sleeping under treated net previous night) | 0.89 (0.75 – 1.05) | 0.166) | 0.53 (0.43 – 0.65) | <0.001 |
| **Educational attainment**  (Post primary, primary, None) | 0.85 (0.81 – 0.88) | <0.001 | 0.88 (0.85 – 0.91) | <0.001 |
| **Nutritional status**^1^  (1= No Anaemia, 0 =Anaemia) | 0.53 (0.42 – 0.66)^1^ | < 0.001 | 0.65 (0.51 – 0.82)^1^ | <0.001 |

^1^ Assessed among 6 months to 15-year-olds (Rainy season; N = 1606, dry season; N =1,841); In actual mediation analysis, we used LLIN use and not LLIN coverage.

1. Third criteria are that change in levels of exposure significantly changes the outcome. This has been critiqued by many scholars including Mackinnon 2008. Consensus has now been reached that the relationship between A and Y need not be statistically significant for M to be a mediator. Lack of significant association between X and Y could be due to suppression. Suppression happens when a mediating effect of a competing process has the opposite sign of mediating effect of interest
2. When the previously defined paths are controlled, a previously significant relation between the exposure and outcome is no longer significant, with the strongest demonstration of mediation occurring when the path from the independent variable to the outcome variable is zero. This is no longer correct because there is possible partial mediation (i.e., there are potentially other mediators not considered). In any case, the change from significant to non-significant after adjusting for mediator may be just a trivial change for example p-value change from 0.049 to 0.051. Whether this small change is indicative of mediation is questionable.

- *Based on this approach; Housing, educational attainment and nutritional (both seasons), and LLIN use (dry season) would be considered strong mediator candidates.*
- *This approach has fundamental flaws and we only use it as explorative analysis. For mediation, we used modern methods - counterfactual approach to mediation which we applied in this study.*
