# Supplementary material for: Socioeconomic disparities in Plasmodium falciparum infection risk in Southern Malawi: mediation analyses
Source: Sci Rep. 2024 Nov 8;14:27290. doi: 10.1038/s41598-024-78512-1 (PMC11549479; doi:10.1038/s41598-024-78512-1)
Supplement: Supplementary file 5 — Supplementary Material 5 [file 41598_2024_78512_MOESM5_ESM.docx]

**Additional file 5. Mediation analysis results by different subgroups in different seasons**

**Table 5a: Total Effect (TE), Total Natural Direct Effect (TNDE), and Total Natural Indirect Effect (TNIE) on the association between SEP and** *Pf infection***, with a breakdown of proportions mediated by different pathways (Rainy season)**

|  | **6 months to 5 years (N= 552)** | | **5-15 years (N= 1,106)** | | | **Adults, 16+ (N= 1,339)** | |
| --- | --- | --- | --- | --- | --- | --- | --- |
| **Mediators** | Estimate  (95% CI) | E. Values for unmeasured confounding, RR [CI] | Estimate (95% CI) | E. Values for unmeasured confounding (confounding, RR [CI] | | Estimate (95% CI) | E. Values for unmeasured confounding, RR [CI] |
| **Housing quality** |  |  |  |  | |  |  |
| Total effect (of SEP) | 0.22 (0.09 – 0.43) | 8.57 [4.04] | 0.26 (0.20 – 0.34) | 7.21 [5.30] | | 0.43 (0.35 – 0.59) | 4.05 [2.76] |
| TNDE | 0.49 (0.19 – 0.88) | 3.54 [1.52] | 0.49 (0.31 – 0.84) | 3.48 [1.69] | | 0.72 (0.64 – 0.88) | 2.12[1.51] |
| TNIE | 0.44 (0.40 – 0.50) | **3.90 [3.41]** | 0.68 (0.55 – 0.77) | **3.28 [2.46]** | | 0.60 (0.51– 0.69) | **2.72 [2.27]** |
| Proportion mediated | **34.8%** |  | **32.5%** |  | | **51.0%** |  |
| **Highest educational attainment (post primary)** |  |  |  |  | |  |  |
| TNDE | 0.37 (0.12 – 0.69) | 4.85 [2.28] | 0.36 (0.31 – 0.49) | 5.00 ]3.50] | | 0.54(0.53 – 0.76) | 3.12 [1.98] |
| TNIE | 0.61 [0.55– 0.71) | **2.68 [2.16]** | 0.71 (0.65 – 0.86) | **2.19 [1.59]** | | 0.80 (0.73 – 0.84) | **1.81 [1.66]** |
| Proportion mediated | **18.7%** |  | **14.3%** |  | | **19.0%** |  |
| **Food security (secure)** |  |  |  |  | |  |  |
| TNDE | 0.24 (0.10 – 0.44) | 7.64 [3.88] | 0.26 (0.21 – 0.37) | 7.03 [4.84] | | 0.43 (0.35 – 0.51) | 4.05 [3.34] |
| TNIE | 0.92 (0.88 – 1.02) | 1.41 [1.00] | 0.98 (0.93 – 0.99) | **1.18 [1.08]** | | 0.99 (0.99 – 1.01) | 1.02 [1.00] |
| Proportion mediated | 2.6% |  | **1.0%** |  | | 0.0% |  |
| **LLIN use** |  |  |  |  | |  |  |
| TNDE | 0.23 (0.09 – 0.45) | 8.32 [3.89] | 0.25 (0.21 – 0.35) | 7.21 (5.22) | | 0.43 (0.35 – 0.60) | 4.03 [2.74] |
| TNIE | 0.99 (0.99 – 1.03) | 1.06 [1.00] | 1.00 (0.99 – 1.01) | 1.05 (1.00) | | 0.99 (0.98 – 0.99) | 1.11 [1.02] |
| Proportion mediated | 0.1% |  | -0.01% |  | | **0.1%** |  |
| **Nutritional status (No anemia)** |  |  |  |  | |  |  |
| TNDE | 0.25 (0.09 – 0.49) | 7.32 [3.52] | 0.34 (0.18 – 0.39) | 5.33 [4.57] | |  |  |
| TNIE | 0.91 (0.88 – 0.97) | **1.43 [1.20]** | 0.75 (0.70 – 0.79) | **2.00 [1. 84]** | |  |  |
| Proportion mediated | **3.0%** |  | **11.6%** |  | |  |  |
| **Combined significant mediators** | |  |  | |  |  |  |
| TNDE | 0.90 (0.73 – 1.76) | 1.45 [1.00] | 0.77 (0.67 – 1.19) | 1.91 [1.00] | | 0.88 (0.51 – 1.17) | 1.51 [1.00] |
| TNIE | 0.25 (0.18 – 0.35) | **7.34 (5.09]** | 0.33 (0.29 – 0.43) | **5.51 [4.78]** | | 0.49 (0.38 – 0.58) | **3.51 [2.88]** |
| Proportion mediated | **87.9%** |  | **70.3%** |  | | **80.0% ^*^** |  |

**^*^**Exposure mediator interactions present and considered.

***Interpretation:*** *The indirect/mediated’ effects (through housing, education and LLIN use) are consistently significant across the different age groups. However, the proportion mediated varies considerably with large effects among adults followed by under 5 and smaller effects among the school-age children. Good nutritional status was an important mediator for under 5 and school age children (5 – 15 years). Mediated effect through nutritional status needs to be interpreted cautiously in light of bidirectional relationship between malaria and anemia that can’t be distinguished due to design limitations.*

**Table 5b: Total Effect (TE), Total Natural Direct Effect (TNDE), and Total Natural Indirect Effect (TNIE) on the association between SEP and** *Pf infection***, with a breakdown of proportions mediated by different pathways (Dry season).**

|  | **6 months to 5 years (N= 595)** | | **5-15 years (N= 1,272)** | | **Adults, 16+ (N= 1,379)** | |
| --- | --- | --- | --- | --- | --- | --- |
| **Mediators** | Estimate  (95% CI) | E. Values for unmeasured confounding, RR [CI] | Estimate (95% CI) | E. Values for unmeasured confounding, RR [CI] | Estimate (95% CI) | E. Values for unmeasured confounding, RR [CI] |
| **Housing quality** |  |  |  |  |  |  |
| Total effect | 0.22 (0.20 – 0.32) | 8.56 [5.70] | 0.21 (0.13 – 0.24) | 9.04 [7.61] | 0.21 (0.08 – 0.27) | 8.99 [6.89] |
| TNDE | 0.34 (0.34 – 0.61) | 5.33 [2.66] | 0.34 (0.17 – 0.42) | 5.31 [4.14] | 0.31 (0.13 – 0.44) | 5.90 [3.97] |
| TNIE | 0.64 (0.48 – 0.69) | **2.50 [2.25]** | 0.61 (0.54 – 0.75) | **2.68 [1.98]** | 0.66 (0.59 – 0.69) | **2.39 [2.26]** |
| Proportion mediated | **15.5%** |  | **17.1%** |  | **13.6%** |  |
| **Highest educational attainment (post primary)** | |  |  |  |  |  |
| TNDE | 0.42 (0.30 – 0.73) | 4.21 [2.07] | 0.32 (0.16 – 0.46) | 5.79 [3.81] | 0.23 (0.15 – 0.37) | 8.12 [4.80] |
| TNIE | 0.54 (0.39 – 0.64) | **3.13 [2.50]** | 0.66 (0.59 – 0.77) | **2.38 [1.92]** | 0.93 (0.87 – 1.02) | 1.37 [1.00] |
| Proportion mediated | **24.8%** |  | **13.3%** |  | 2.2% |  |
| **Food security (secure)** | |  |  |  |  |  |
| TNDE | 0.21 (0.14 – 0.35] | 9.08 [5.11] | 0.21 (0.11 – 0.27) | 8.87 [6.63] | 0.20 (0.09 – 0.29) | 9.47 [6.35] |
| TNIE | 1.03 [0.99 – 1.04} | 1.24 [1.00] | 0.98 (0.90 – 1.01) | 1.16 [1.00] | 1.02 (0.96 – 1.08) | 1.16 [1.00] |
| Proportion mediated | -0.2 |  | 0.0% |  | -0.1% |  |
| **LLIN use** |  |  |  |  |  |  |
| TNDE | 0.25 (0.14 – 0.36) | 7.46 [4.70] | 0.26 (0.17 – 0.29) | 7.09 [6.29] | 0.21 (0.08 – 0.25) | 9.22 [7.49] |
| TNIE | 0.83 (0.83 – 0.96] | **1. 70 [1.25]** | 0.80 (0.76 – 0.86) | **1.80 [1.58]** | 1.02 (0.97 – 1.10) | 1.17 [1.00] |
| Proportion mediated | **5.4%** |  | **6.5%** |  | -0.1% |  |
| **Nutritional status (No anemia)** | |  |  |  |  |  |
| TNDE | 0.22(0.16 – 0.26) | 8.40 [5.00] | 0.25 (0.13 – 0.31) | 7.60 [5.86] |  |  |
| TNIE | 0.96 (0.94 – 1.01) | 1.24 [1.00] | 0.85 (0.83 – 0.90) | 1.62 [1.47] |  |  |
| Proportion mediated | 1.1% |  | **4.6%** |  |  |  |
| **Combined mediators** | |  |  |  |  |  |
| TNDE | 0.60 (0.16 – 0.89) | 2.71 [1.47] | 0.58 (0.33– 0.78) | 2.87 [1.88] | 0.31 (0.13 – 0.44) | 5.90 [3.97] |
| TNIE | 0.36(0.31 – 0.51) | **5.01 [3.31]** | 0.35 (0.31 – 0.45) | 5.21 [3.83] | 0.66 (0.59 – 0.69) | **2.39 [2.26]** |
| Proportion mediated | **49.5%** |  | **47.6%** |  | **13.6%** |  |

***Interpretation.*** *Housing is a mediator at all ages contributing more among adults, followed by children under 5 and least among school-aged children****.*** *Housing is the only mediator among adults (16+). Educational attainment, and LLIN use were important mediators among both children under 5 and school-aged children (5-15 years). Good nutrition appears to mediate a small proportion of the effect of SEP on Pf infection only among school-aged children. Mediated effect through nutritional status needs to be interpreted cautiously in light of bidirectional relationship between malaria and anemia that can’t be distinguished due to design limitations.*
