# Supplementary material for: Socioeconomic disparities in Plasmodium falciparum infection risk in Southern Malawi: mediation analyses
Source: Sci Rep. 2024 Nov 8;14:27290. doi: 10.1038/s41598-024-78512-1 (PMC11549479; doi:10.1038/s41598-024-78512-1)
Supplement: Supplementary file 6 — Supplementary Material 6 [file 41598_2024_78512_MOESM6_ESM.docx]

**Additional file 6: Distribution of downstream mediators by educational attainment**

|  | **Dry season (Sept/Oct, 2014)**  **(N = 3,223)** | | | **Rainy season (Sept/Oct, 2014)**  **(N = 2,988)** | | |
| --- | --- | --- | --- | --- | --- | --- |
| Characteristic | None,  N = 601 | *Primary,*  N = 1896 | Post primary,  N = 731 | None,  N = 605 | *Primary,*  N = 1,668^1^ | Post primary,  N = 715 |
| Housing quality |  |  |  |  |  |  |
| Low | 424(70.5%) | 933(49.2%) | 136 (18.6%) | 402(66.4%) | 775(46.5%) | 176 (24.6%) |
| High | 177(29.5%) | 963 (50.8%) | 595 (81.4%) | 203(33.6%) | 893(53.5%) | 539 (75.4%) |
| Food security |  |  |  |  |  |  |
| Less secure | 102(17.6%) | 247 (17.3%) | 47 (6.4 %) | 106(17.5%) | 299(17.9%) | 88 (12.3%) |
| Secure | 478(82.4%) | 1,623(86.8%) | 684 (93.6%) | 499(82.5%) | 1,369(82.1%) | 626 (87.7%) |
| Net use (slept in net previous night) | |  |  |  |  |  |
| No | 404(67.2%) | 1,112(59.2%) | 319 (43.6%) | 254(42.0%) | 579 (34.7%) | 218 (30.5%) |
| Yes | 197(32.8%) | 774 (40.8%) | 412 (56.4%) | 351(58.0%) | 1089 (65.3%) | 497 (69.5%) |
| Nutritional status (6 months – 15 years) | |  |  |  |  |  |
| Had anemia | 182(57.1%) | 560 (50.7%) | 176 (43.5%) | 195(60.9%) | 506 (56.4%) | 174 (45.4%) |
| No Anemia | 13(42.9%) | 545 (49.3%) | 229 (56.5%) | 125(39.1%) | 391 (43.6%) | 209 (54.6%) |

*^*^The table indicates differential distribution of other mediators by educational attainment. Educational attainment which is more upstream mediator appears to correlate (have influence) on more proximal potential mediators of the association between SEP and plasmodium falciparum infection*
